# FOXC2 as a prognostic marker and a potential molecular target in patients with human solid tumors

**DOI:** 10.3389/fsurg.2022.960698

**Published:** 2022-11-08

**Authors:** Long Zhang, Yong He, Xiaohong Tu, Chao Wang, Xiaojun Ding, Rongqiang Ye, Jiayu Shi, Yuancai Xie, Yufen Jiang, Xiaohong Deng

**Affiliations:** ^1^ Department of Hepatopancreatobiliary Surgery, Ganzhou People's Hospital of Jiangxi Province (Ganzhou Hospital Affiliated to Nanchang University), Ganzhou, China; ^2^ Department of Physical Education, Ganzhou Teachers College, Ganzhou, China; ^3^ Hepatic Surgery Center, Institute of Hepato-pancreato-biliary Surgery, Department of Surgery, Tongji Hospital, Tongji Medical College, Huazhong University of Science and Technology, Wuhan, China; ^4^ Department of Hepato-pancreato-biliary Surgery, Wuhan University of Science and Technology, Wuhan, China; ^5^ Department of Gastroenterology, Kezhou People’s Hospital, Atushi, China

**Keywords:** FOXC2, solid tumor, prognosis, survival, tumor biomarker

## Abstract

**Background:**

Forkhead Box Protein C2 (FOXC2) belongs to the Forkhead/Wing-helix family. The regulatory role of this transcription factor in physiological function and carcinogenic activity has been proven in subsequent investigations. However, there is still scarcity of evidence on the relationship between FOXC2 expression and prognosis in human solid tumors. We conducted this meta-analysis to evaluate the role of FOXC2 as a prognosis factor and a possible target marker in human solid tumors.

**Methods:**

PubMed, Web of Science, Embase, and the Cochrane library database were all searched methodically. Eligible publications on FOXC2 in human solid tumors were gathered and reviewed. The effect sizes were calculated using pooled hazard ratios (HRs) or odds ratios (ORs) with the corresponding 95% confidence interval (CI). Statistical analysis was conducted with Stata SE12.0.

**Results:**

This meta-analysis comprised 3,267 patients from 20 studies covering a variety of solid tumors. Increased FOXC2 expression was related to shorter overall survival (OS) (HR = 2.05, 95% CI: 1.73–2.42). High expression of FOXC2 is associated with lymph node metastases (OR = 3.33, 95% CI: 2.65–4.19), TNM stage (OR = 3.09, 95% CI: 2.00–4.78), and age (OR = 1.26, 95% CI: 1.06–1.50), according to the pooled ORs. However, no significant association was observed between the high expression of FOXC2 and sex, tumor size or tumor differentiation.

**Conclusion:**

Increased expression of FOXC2 is associated with unfavored OS, lymph node metastases, TNM stage, and age. FOXC2 is a promising prognostic marker and a novel target marker in human solid tumors.

## Introduction

The transcription factor forkhead box (FOX) is a family with a highly conserved winged-helix DNA-binding domain (1, 2). FOX family members are involved in cell growth, differentiation, aging and carcinogenesis, and various regulatory and functional activities (3, 4). From FOXA to FOXR, there are 17 gene subfamilies of FOX and more than 14 have been identified in humans (5). FOXC2, also known as the mesenchyme forkhead-1 (MHF1), consists of a single exon located on the chromosomal band 16p24.1 (6). FOXC2 is necessary for the development of the lungs (7), bone (8), cardiovascular system (9), adipose tissue in adults (10), and various other organs or tissues. In addition to physiologic functions as cellular metabolism, angiogenesis and wound healing, dysregulated FOXC2 contributes to tumorigenesis and malignancy progression in cell proliferation, metabolic reprogramming, lymph-angiogenesis, epithelial-mesenchymal transition (EMT), and drug resistance (11–16). Recent studies have reported that FOXC2 is dysregulated in malignancies, including breast cancer (13), gastric cancer (17), esophageal carcinoma (18).

Currently, there is an increasing interest on the oncogenic role of FOXC2 both *in vivo* and *in vitro*. FOXC2 has also merged as a potential molecular target in preclinical/clinical studies due to the dysregulated expression level and nuclear localization. Previous studies have associated expression levels of FOXC2 with clinical and pathological characteristics including tumor size, differentiation, metastasis, and stage (19, 20). However, there is still lack of proof that FOXC2 expression in human solid tumors has significant predict value. This analysis was carried out in order to systematically assess the potential prognostic significancy of FOXC2.

## Methods

### Literature search

A systematic literature search was undertaken in PubMed, Web of Science, Embase, and Cochrane library databases before April, 2022. The following keywords and search terms were used to find potentially eligible studies: (“Forkhead box protein C2” OR “*FOXC2*” OR “*MHF1*” OR “mesenchyme forkhead1”) AND (“cancer” OR “carcinoma” OR “neoplasm” OR “tumor” OR “malignancy”) AND (“prognosis” OR “survival”). Additional research was found by looking through the references of the selected articles. The Preferred Reporting Items for Systematic reviews and Meta-Analyses (PRISMA) statement was used in this analysis (21).

### Study selection

The following were the selection criteria of this analysis: (1) patients with solid tumors diagnosed pathologically; (2) the expression FOXC2 in tissue were determined by immunohistochemistry (IHC) or quantitative real-time polymerase chain reaction (q-PCR); (3) available data for calculating odds ratio (OR) with 95% CIs were depicted; (4) only the study with the most extensive or recent data was considered, if multiple publications used employed overlapping samples from the same institution; (5) patients were categorized into groups based on high and low FOXC2 expression levels. Exclusion criteria in this meta-analysis were as follows: (1) duplicate publications; (2) research with no data or data from animal or cellular experiments; (3) only serum levels of FOXC2 expression were detected; (4) studies only provided Kaplan-Meier curves but no multivariate data; (5) reviews, letters, case reports, or expert opinions.

### Date extraction and quality assessment

The two independent investigators screened the all publications, classified and sorted out the titles and abstracts of the literature retrieved from reading, excluded duplicate literatures and literature failed inclusion criteria, contacted the original author for relevant information for the literature with incomplete information report, and determined whether it could be included in the final study after obtaining the full text. The research team shall assist in solving any dispute. The following information was retrieved from eligible articles: name of first author; publication year; sample size; cancer kind; criteria for increased expression of FOXC2; detecting methodology; outcome measuring; patient follow-up; HRs with corresponding 95% CI; and clinical characteristics (age, sex, tumor size, lymph node metastases, distant metastases, TNM stage). We preferred multivariate analysis in research with both univariate and multivariate analyses because it is better at explaining confounding factors. If there was a disagreement, a compromise was sought through debate until everyone agreed. The quality evaluation for eligible studies was undertaken by 2 independent investigators (CW and LZ), and any discrepancies were handled by consensus among all authors. The Newcastle-Ottawa Scale (NOS) tool was used to evaluate the quality of all eligible studies (22). The NOS scores ranged from 0 to 9. High quality was assigned to studies with NOS score ≥ 6.

### Statistical analysis

Stata SE12.0 was applied to conduct this meta-analysis (Stata Corp., College Station, USA). The heterogeneity of the included studies used Chi-square-based Q test and *I*^2^ statistic (23). *P* < 0.05 for the Q test and an *I*^2^ > 50% indicates significant heterogeneity. For studies with no obvious heterogeneity (*P*_h_ > 0.05, *I*^2^ < 50%), the fixed-effects model was adopted, and the random-effects model was used for others (*P*_h_ ≤ 0.05, *I*^2^ ≥ 50%). The sensitivity analysis was conducted to check the stability of results. Begg's and Egger's tests were conducted to investigate potential publication bias (24). Differences with *P* < 0.05 were considered as statistically significant.

## Results

### Study characteristics

**Figure 1:**
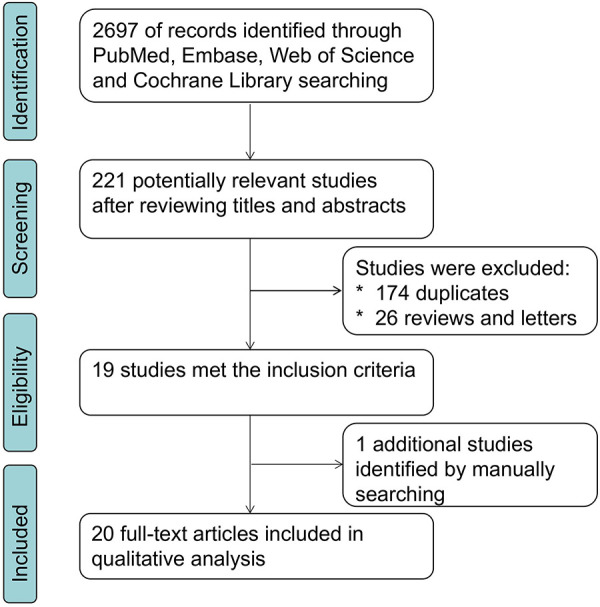
Literature search and selection flowchart.

The procedure of literature retrieval was depicted in Figure 1. A total of 3,267 patients with solid tumors were included in eligible articles published between 2011 and 2021 (17–19, 25–40). These studies were conducted in China (*n* = 12), Japan (*n* = 5), Singapore (*n* = 1), Spain (*n* = 1), and Norway (*n* = 1). Mean of patient sample size was 163 (from 61 to 338). In this meta-analysis, 15 varying solid tumor kinds were summarized, including 2 non–small-cell lung cancer, 2 breast cancers, 2 esophageal squamous cell carcinoma, 2 colorectal cancer, 2 cervical cancer, and 1 each of glioma, oral squamous cell carcinoma, pulmonary neuroendocrine tumors, extrahepatic cholangiocarcinoma, hepatocellular carcinoma, gastric cancer, oral tongue squamous cell carcinoma, ovarian cancer, prostate cancer, and phyllodes tumor of the breast. All of the specimens were well preserved, and diagnosis was made based on pathological findings. The main characteristics of enrolled studies are summarized in Supplementary Table S1. Eligible studies included in this meta-analysis had NOS scores ranging from 5 to 9, with a mean of 6.5.

### Prognostic value of FOXC2 in patients with solid tumors

**Figure 2:**
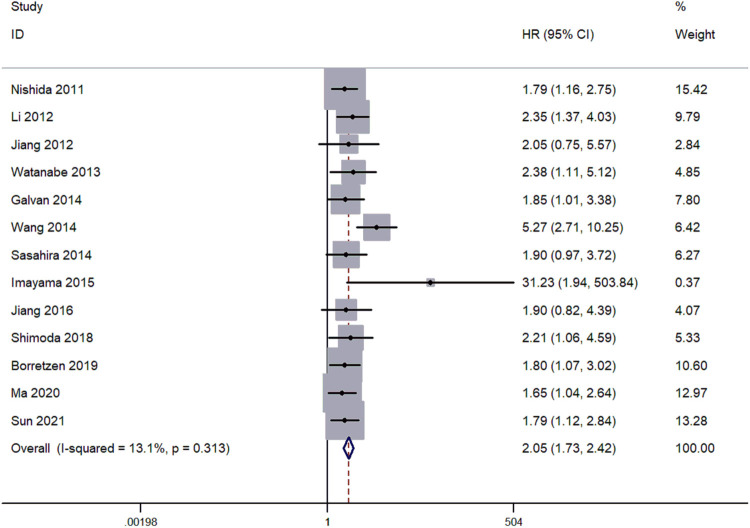
Forest plots for the relationship between high FOXC2 expression and OS.

In 13 articles, the overall survival (OS) was reported. The pooled hazard ratios (HRs) and corresponding 95% CI were estimated by the fixed-effects model. The results indicated a mild heterogeneity in studies (*I*^2^ = 13.1%, *P*_h_ = 0.313). HRs for the increased FOXC2 expression against the low FOXC2 expression were 2.31 (95% CI: 1.73–2.42) (Figure 2). Patients with increased expression of FOXC2 presented significantly shorter OS, indicating that increased FOXC2 expression was associated with unfavored OS.

### Clinical and pathological characteristics associated with FOXC2 expression

**Figure 3:**
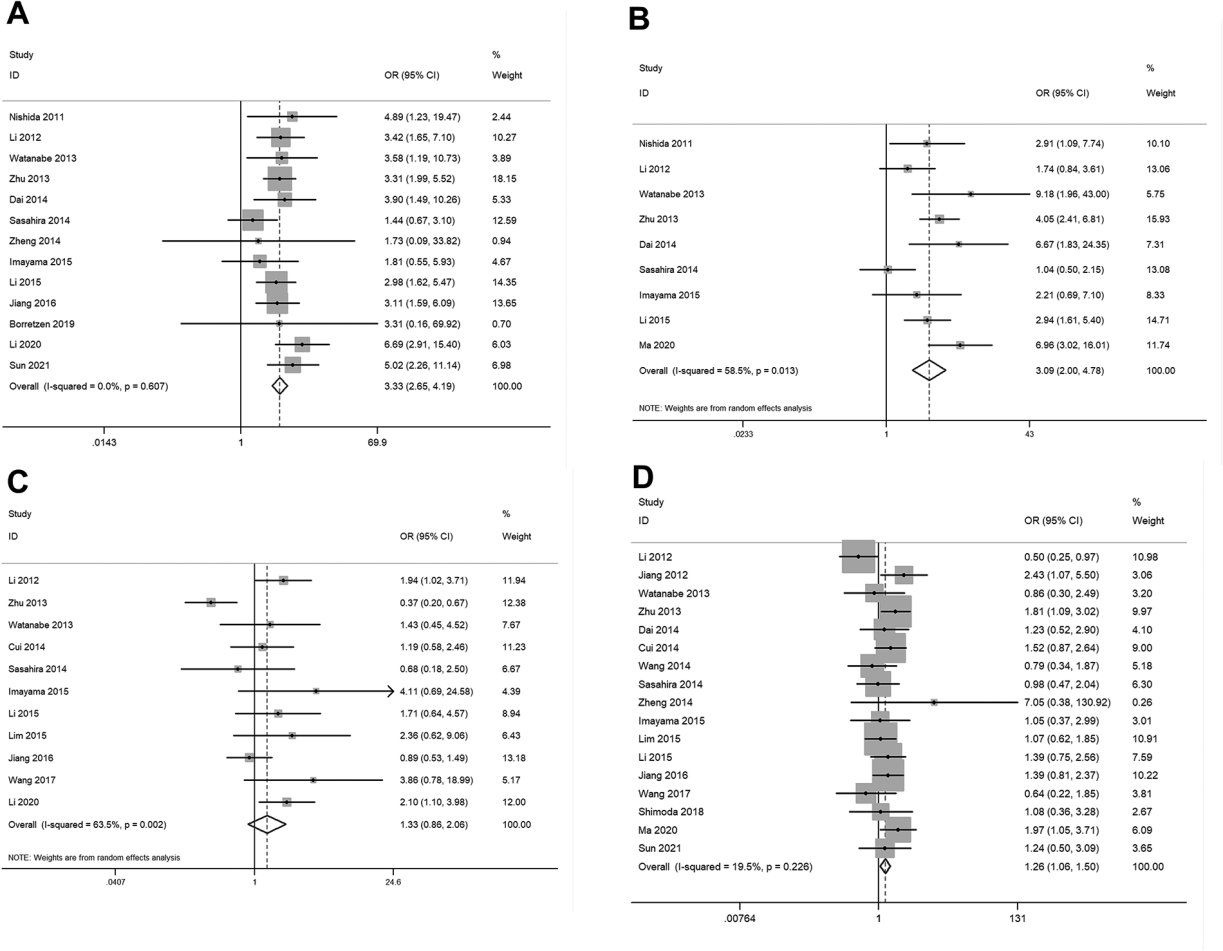
Forest plots for the relationship between FOXC2 overexpression and clinical and pathological characteristics. (**A**) Lymph node metastases, (**B**) TNM stage, (**C**) tumor differentiation, (**D**) patient age.

The pooled results (Supplementary Table S2) showed that elevated expression of FOXC2 was significantly related with lymph node metastases (OR = 3.33, 95% CI: 2.65–4.19, *P *< 0.05) (Figure 3A), TNM stage (OR = 3.09, 95% CI: 2.00–4.78, *P* < 0.05) (Figure 3B), and age (OR = 1.26, 95% CI: 1.06–1.50, *P* < 0.05) (Figure 3D). However, no significant correlation was observed between increased expression of FOXC2 and tumor differentiation (Figure 3C), sex, or tumor size (data not shown). Due to a lack of data, we were unable to detect the relationship between FOXC2 overexpression and other clinical and pathological characteristics.

### Sensitivity analysis

**Figure 4:**
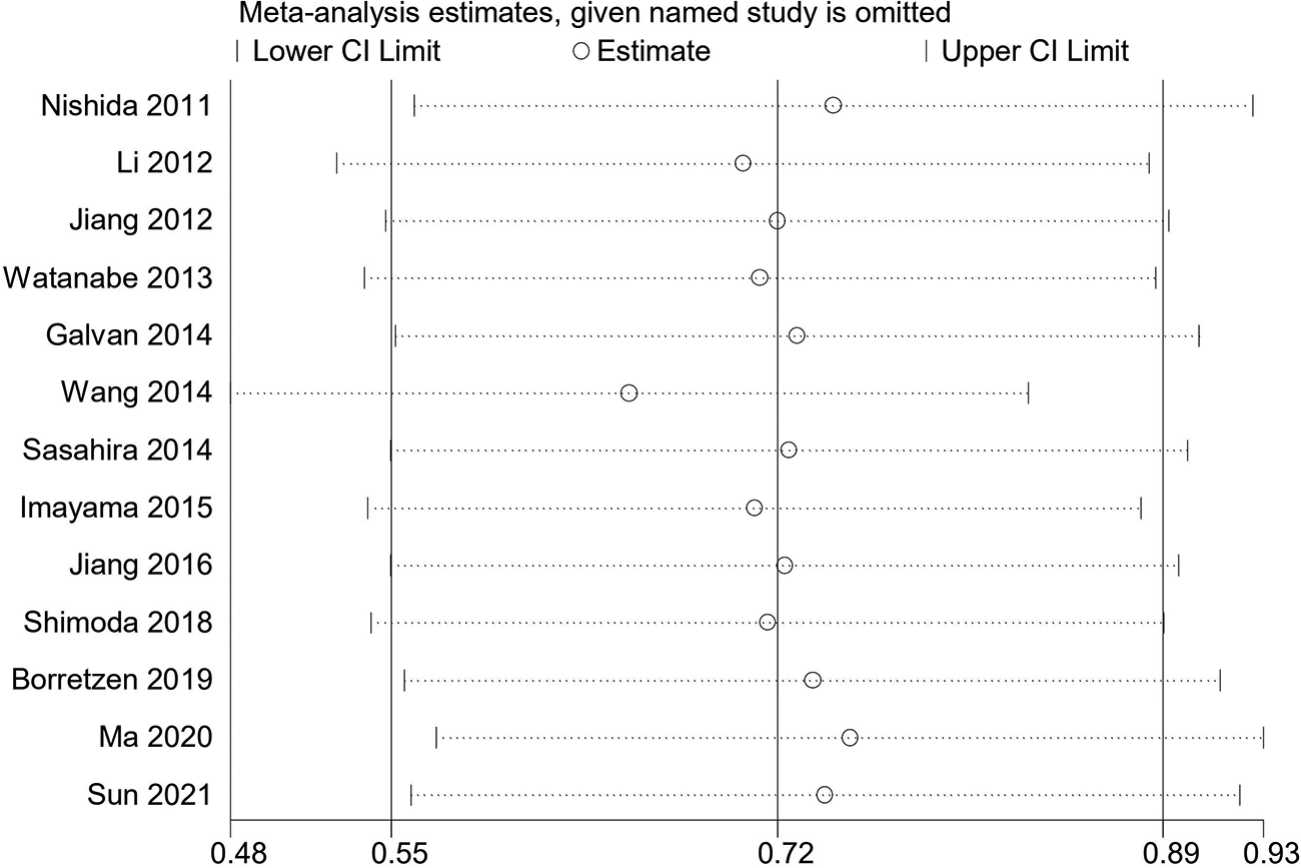
Sensitivity analysis regarding overall survival.

Sensitivity analysis was conducted to assess the FOXC2 expression and OS by gradually deleting each individual research from the pooled analysis. The purpose of this approach is to evaluate the impact of the deleted data set on the overall HRs. The fingdings were reliable, and the exclusion of any study had no effect on the results (Figure 4).

### Publication bias

**Figure 5:**
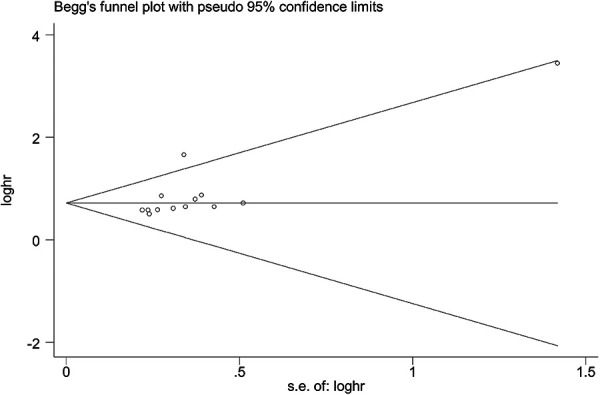
Publication bias in this meta-analysis.

Begg's Test and Egger's tests were conducted to evaluate the publication bias. Findings revealed that there was no publication bias between FOXC2 expression and OS in the included studies (Figure 5).

## Discussion

As a kind of genomic disease, lots of somatic mutations, structural mutations and gene recombination occur during the carcinogenesis process (41). There were 19.3 million new cancer cases and 10.0 million cancer deaths estimated in 2020 worldwide, with cancer burden anticipated to rise to 28.4 million by 2040 (42). Despite advancements in cancer surveillance enrollment, surgical techniques, systematic therapy and palliative care, the survival of individuals with solid tumors still remains unsatisfactory. Finding novel tumor markers is critical in order to provide accurate diagnosis and prospective therapeutic targets.

FOXC2 acts as a key mediator of tumor initiation and progression, involving tumor proliferation, migration, invasion, metastasis, and EMT (3). FOXC2 overexpression has been reported in a range of tumor kinds, including lung cancer (19), colorectal cancer (15), gastric cancer (16), ovarian cancer (40) and glioma (32). Furthermore, FOXC2 overexpression was associated with clinical characteristics and a poor prognosis (43, 44). FOXC2 is a novel independent biological marker for predicting tumor progression and survival because of its prognostic significancy and association with clinicopathological features. The prognosis effect of elevated FOXC2 expression was assessed in patients with solid tumors. The findings indicated that elevated FOXC2 expression was related with shorter OS in solid tumors. Additionally, increased FOXC2 was closely associated with age, TNM stage and lymph node metastasis, suggesting that FOXC2 could be a useful biomarker for predicting prognosis in human solid tumors based on clinical pathology. Targeting FOXC2 might be a viable approach for these patients.

The limitations of this analysis were as follows: first, in this meta-analysis, the majority of the studies were conducted among Asian population. Other ethnic groups, such as Europeans, Africans and Americans, are relatively under-studied, which may limit the global applicability of the results discussed. Further high-quality studies from diverse ethnical origins are necessary to investigate the therapeutic importance of FOXC2. Second, despite the fact that FOXC2 overexpression was associated with patient age, tumor differentiation, lymph node metastasis and TNM stage, we were unable to evaluate the association between FOXC2 overexpression and other clinical and pathological characteristics due to insufficient data. Third, the number of studies included in this analysis could restrict its statistical power. Although no publication bias was found, potential publication bias may still exist due to the insufficient studies available for assessments. Then, inconsistencies in detecting platforms, methodologies, and criteria for IHC or RT-PCR, and distinct tumor kinds with varying prognostic differences may lead to skewed results. Furthermore, the mean of NOS scores is 6.5, implying that the quality of studies in this analysis is acceptable but not supreme, which might be inevitable in meta-analysis. Finally, the combined predictive significance of FOXC2 and other tumor markers was not assessed. As a result, higher quality multicenter studies with larger population, as well as consistent criteria for assessing the expression of FOXC2, are necessary for validation of the findings.

## Conclusion

In this analysis, increased expression level of FOXC2 is associated with poor prognosis, as well as TNM stage, lymph node metastases, and age. FOXC2 could serve as a novel prognostic marker in solid tumors. For these patients, targeting FOXC2 could be a feasible treatment option. To corroborate the findings, further well-designed pre-clinical/clinical studies with high-quality data are needed.

## Data Availability

The raw data supporting the conclusions of this article will be made available by the authors, without undue reservation.
