# Supplementary material for: FOXC2 as a prognostic marker and a potential molecular target in patients with human solid tumors
Source: Front Surg. 2022 Nov 8;9:960698. doi: 10.3389/fsurg.2022.960698 (PMC9679010; doi:10.3389/fsurg.2022.960698)
Supplement: Supplementary file 1 [file Table1.doc]

| **Supplemental Table 1.** Main characteristics of studies included in this analysis | | | | | | | | | | |
| --- | --- | --- | --- | --- | --- | --- | --- | --- | --- | --- |
| **First author** | **Publication year** | **Cancer type** | **Total number** | **Tumor stage** | **Follow up  (years)** | **Detection method** | **Outcome  measure** | **Multivariate analysis** | **Treatment received** | **Study type** |
| Nishida et al (18) | 2011 | ESCC | 70 | 39/31 (I-II/III-IV) | >10 years | RT-PCR | OS | Yes | S | Prospective study |
| Li et al (28) | 2012 | BC | 262 | 152/110 (I-II/III-IV) | >6 years | IHC | OS+DFS | Yes | S | Prospective study |
| Jiang et al (27) | 2012 | NSCLC | 137 | 137/0 (I-II/III-IV) | >4 years | IHC | OS+RFS | Yes | S | Prospective study |
| Watanabe et al (34) | 2013 | EHCC | 77 | 31/49 (I-II/III-IV) | >5 years | IHC | OS | Yes | S | Prospective study |
| Zhu et al (17) | 2013 | GC | 325 | 148/177 (I-II/III-IV) | >9 years | IHC | NR | No | S | Prospective study |
| Wang et al (32) | 2014 | gliomas | 85 | 28/57 (I-II/III-IV) | >4 years | IHC | OS | Yes | S | Prospective study |
| Sasahira et al (31) | 2014 | OSCC | 163 | 87/76 (I-II/III-IV) | >10 years | IHC | OS | Yes | S | Prospective study |
| Galvan et al (38) | 2014 | PNETs | 134 | NR | >5 years | IHC | OS | Yes | S+C+R | Retrospective study |
| Dai et al (25) | 2014 | BC | 103 | 91/12 (I-II/III-IV) | NR | IHC | NR | No | S | Retrospective study |
| Cui et al (43) | 2014 | CRC | 206 | NR | >8 years | IHC | NR | No | S | Prospective study |
| Zheng et al (35) | 2014 | CC | 69 | 69/0 (I-II/III-IV) | >7 years | IHC | NR | No | S | Retrospective study |
| Imayama et al (26) | 2015 | OTSCC | 61 | 45/16 (I-II/III-IV) | >5 years | IHC | OS | Yes | S | Retrospective study |
| Lim et al (30) | 2015 | PTs | 271 | NR | >8 years | IHC | NR | No | S | Retrospective study |
| Li et al (29) | 2015 | CRC | 185 | 105/80 (I-II/III-IV) | >6 years | IHC | NR | No | S | Prospective study |
| Jiang et al (19) | 2016 | NSCLC | 309 | 62/247 (I/II-III) | >5 years | IHC | OS+RFS | Yes | S+C+R | Prospective study |
| Wang et al (33) | 2017 | CC | 66 | 30/36 (I-II/III-IV) | >2 years | IHC | NR | No | S | Prospective study |
| Shimoda et al (36) | 2018 | HCC | 84 | NR | >10 years | IHC | OS | Yes | S | Prospective study |
| Borretzen et al (37) | 2019 | PC | 338 | NR | >10 years | IHC | OS | Yes | S+C+H | Retrospective study |
| Ma et al (39) | 2020 | ESCC | 170 | 143/27 (I-II/III-IV) | >5 years | IHC | OS | Yes | S | Retrospective study |
| Sun et al (40) | 2021 | OC | 153 | 10/72 (I-II/III-IV) | >2 years | IHC | OS | Yes | S | Retrospective study |
| IHC, immunohistochemistry; RT-PCR, quantitative real-time polymerase chain reaction; BC, breast cancer; CRC, colorectal cancer; ESCC, esophageal squamous cell carcinoma; GC, gastric cancer; HCC, hepatocellular carcinoma; NSCLC, non-small-cell lung cancer; OSCC, oral squamous cell carcinoma; PNETs, pulmonary neuroendocrine tumors; CC, cervical cancer; OTSCC, oral tongue squamous cell carcinoma; OC, ovarian cancer; PC, prostate cancer; OS, overall survival; S, surgery; C, chemotherapy; R, radiotherapy; NR, not reported. | | | | | | | | | | |
