# Supplementary material for: FOXC2 as a prognostic marker and a potential molecular target in patients with human solid tumors
Source: Front Surg. 2022 Nov 8;9:960698. doi: 10.3389/fsurg.2022.960698 (PMC9679010; doi:10.3389/fsurg.2022.960698)
Supplement: Supplementary file 2 [file Table2.docx]

| **Supplemental Table 2.** The relationship of FOXC2 expression and clinicopathological parameters. | | | | | | | |
| --- | --- | --- | --- | --- | --- | --- | --- |
| **Clinicopathological parameter** | **Studies (n)** | **Patients (n)** | **OR (95% CI)** | **P value** | **I^2^ (%)** | **P_h_** | **Model** |
| Age (big vs. small) | 18 | 2673 | 1.26 (1.06-1.50) | *0.009* | 19.5 | 0.226 | Fixed-effects |
| Sex (male vs. female) | 11 | 1802 | 1.12 (0.91-1.38) | *0.286* | 0 | 0.754 | Fixed-effects |
| Lymph node metastasis (yes vs. no) | 13 | 2145 | 3.33 (2.65-4.19) | *<0.001* | 0 | 0.607 | Fixed-effects |
| TNM stage (III-IV vs. I-II) | 9 | 1419 | 3.09 (2.00-4.78) | *<0.001* | 58.5 | 0.013 | Random-effects |
| Tumor differentiation (poor vs. well/moderate) | 11 | 2092 | 1.33 (0.86-2.06) | *0.204* | 63.5 | 0.002 | Random-effects |
| Tumor size (big vs. small) | 8 | 1239 | 1.31 (0.83-2.05) | *0.248* | 56.8 | 0.023 | Random-effects |
